# Longitudinal analysis of post-acute chikungunya-associated arthralgia in children and adults: A prospective cohort study in Managua, Nicaragua (2014–2018)

**DOI:** 10.1371/journal.pntd.0011948

**Published:** 2024-02-28

**Authors:** Colin M. Warnes, Fausto Andres Bustos Carrillo, Jose Victor Zambrana, Brenda Lopez Mercado, Sonia Arguello, Oscarlette Ampié, Damaris Collado, Nery Sanchez, Sergio Ojeda, Guillermina Kuan, Aubree Gordon, Angel Balmaseda, Eva Harris

**Affiliations:** 1 Division of Infectious Diseases and Vaccinology, School of Public Health, University of California, Berkeley, Berkeley, California, United States of America; 2 Division of Epidemiology and Biostatistics, School of Public Health, University of California, Berkeley, Berkeley, California, United States of America; 3 Sustainable Sciences Institute, Managua, Nicaragua; 4 Centro de Salud Sócrates Flores Vivas, Ministerio de Salud, Managua, Nicaragua; 5 Department of Epidemiology, School of Public Health, University of Michigan, Ann Arbor, United States of America; 6 Laboratorio Nacional de Virología, Centro Nacional de Diagnóstico y Referencia, Ministerio de Salud, Managua, Nicaragua; Universiti Sains Malaysia, MALAYSIA

## Abstract

Chikungunya can result in debilitating arthralgia, often presenting as acute, self-limited pain, but occasionally manifesting chronically. Little is known about differences in chikungunya-associated arthralgia comparing children to adults over time. To characterize long-term chikungunya-associated arthralgia, we recruited 770 patients (105 0–4 years old [y/o], 200 5–9 y/o, 307 10–15 y/o, and 158 16+ y/o) with symptomatic chikungunya virus infections in Managua, Nicaragua, during two consecutive chikungunya epidemics (2014–2015). Participants were assessed at ~15 days and 1, 3, 6, 12, and 18 months post-fever onset. Following clinical guidelines, we defined participants by their last reported instance of arthralgia as acute (≤10 days post-fever onset), interim (>10 and <90 days), or chronic (≥90 days) cases. We observed a high prevalence of arthralgia (80–95%) across all ages over the study period. Overall, the odds of acute arthralgia increased in an age-dependent manner, with the lowest odds of arthralgia in the 0–4 y/o group (odds ratio [OR]: 0.27, 95% confidence interval [CI]: 0.14–0.51) and the highest odds of arthralgia in the 16+ y/o participants (OR: 4.91, 95% CI: 1.42–30.95) relative to 10–15 y/o participants. Females had higher odds of acute arthralgia than males (OR: 1.63, 95% CI: 1.01–2.65) across all ages. We found that 23–36% of pediatric and 53% of adult participants reported an instance of post-acute arthralgia. Children exhibited the highest prevalence of post-acute polyarthralgia in their legs, followed by the hands and torso – a pattern not seen among adult participants. Further, we observed pediatric chikungunya presenting in two distinct phases: the acute phase and the subsequent interim/chronic phases. Thus, differences in the presentation of arthralgia were observed across age, sex, and disease phase in this longitudinal chikungunya cohort. Our results elucidate the long-term burden of chikungunya-associated arthralgia among pediatric and adult populations.

## Author summary

Upon its emergence in the Americas in late 2013, chikungunya virus spread rapidly, leading to >2 million suspected autochthonous chikungunya cases between 2014–2015. Much of what we know about chikungunya is derived from adult populations, leading to gaps in guidelines to treat pediatric chikungunya cases. To address these gaps, we assembled a large cohort of both pediatric (n = 612) and adult (n = 158) laboratory-confirmed (n = 682) or clinically/epidemiologically probable (n = 88) chikungunya cases from two distinct epidemics in 2014 and 2015 in Managua, Nicaragua, followed these patients over a two-year timeframe, and analyzed chikungunya-associated arthralgia using rigorous statistical approaches. Our analysis demonstrates that the pediatric (0–15 years old [y/o]) population faces a previously unappreciated high burden of post-acute chikungunya-associated arthralgia. Further, we observed that post-acute arthralgia presents differently between pediatric and adult cases (16+ y/o). The difference between the two groups was evident when comparing distribution of polyarthralgia across the body parts and when analyzing the persistence of arthralgia >10 days post-fever onset. Using detailed longitudinal data, our findings provide insight into long-term chikungunya-associated arthralgia across age, sex, body parts, and the different phases of chikungunya. We believe these findings will inform clinical guidelines regarding chikungunya-associated arthralgia across all ages.

## Introduction

Symptomatic infection with chikungunya virus (CHIKV), a mosquito-borne alphavirus, typically presents with high fever, rash, headache, myalgia, and debilitating arthralgia, which can be acute or chronic. The name *chikungunya* is derived from the Kimakonde word *kungunyala*, meaning “to become contorted” [1]. Chikungunya is increasingly recognized as causing mortality, particularly among older persons with chronic underlying conditions [2,3]. Current management protocols call for the use of nonsteroidal and steroidal anti-inflammatory drugs and creams, active physical therapy, and psychological treatment in specific cases [4]. Safety and immunogenicity Phase 1, 2, and 3 clinical trials in 2023 have shown promising results – highlighting recent advances in live-attenuated, inactivated, and virus-like particle-based chikungunya vaccine research [5] and culminating in the first accelerated approval of a live-attenuated CHIKV vaccine by the FDA in late 2023 [6].

Chikungunya is often described as having three phases: acute, post-acute/subacute/interim, and chronic [4]. The acute symptoms last 7–10 days post-symptom onset, per the US Centers for Disease Control and Prevention (CDC) [7]. Acute chikungunya can present nonspecifically, complicating differential diagnosis [8]. Though most clinical manifestations subside after the acute phase, chronic arthralgia has been reported up to 6 years post-infection [9]. Chronic chikungunya is often defined as chikungunya-associated sequelae >3 months post-symptom onset [4,10–13]. The period between acute and chronic phases has been described as the subacute or post-acute period, which we call the *interim phase* [4,10,12,14–17]. Being over 45 years old (y/o) and female are considered risk factors for developing chronic chikungunya-associated symptoms and sequelae, though the underlying etiology is unknown [4,10,14]. Despite recent studies on the short- and long-term presentation of chikungunya in adults [4,7,10,13,18–21], the literature is limited regarding the pediatric experience of chikungunya-associated arthralgia over time [14,15], a particular problem as children are considered a vulnerable population that experiences high morbidity in the acute phase of disease [22,23].

In Managua, Nicaragua, CHIKV was first detected in July 2014 – following the introduction of CHIKV into the Americas in late 2013 [24] – with autochthonous transmission observed in September 2014 [25]. CHIKV caused two epidemics in Managua: a moderately sized epidemic in 2014–2015 and a much larger epidemic in 2015–2016. Both epidemics were caused by the Asian lineage, resulting in 61.1 and 218.1 infections per 1,000 person-years, respectively [23,24,26]. We assessed the presence of arthralgia for >18 months post-fever onset among a cohort of chikungunya cases that was recruited from the Pediatric Dengue Cohort Study (PDCS), a longitudinal cohort of ~3,700 children in Managua, as well as adults who received medical care from the study’s health facility, the Health Center Sócrates Flores Vivas (HCSFV). The PDCS, originally designed to study dengue virus infections in children in Nicaragua, was expanded in 2014 to study CHIKV infections and in 2015 to study Zika virus (ZIKV) infections [22,27,28]. All three viruses are spread by the same vector species *Aedes aegypti* mosquitoes. As CHIKV was first introduced into the study area in 2014, the PDCS captured chikungunya cases without previous immunity to CHIKV that could have affected the presentation and duration of illness.

In this study, we quantified differences between adult and pediatric cases. We also characterized the presentation of arthralgia over time and between the sexes to fill gaps in our knowledge of long-term pediatric arthralgia. Using our cohort, we demonstrate the age dependence of arthralgia occurrence, define the frequency of pediatric arthralgia over time, and describe the characteristics of the acute, interim, and chronic phases of chikungunya.

## Methods

### Ethics statement

This study was approved by the Institutional Review Boards of the University of California, Berkeley, and the Nicaraguan Ministry of Health. Participants aged 18 and older and parents or legal representatives of participating children (0–17 y/o) provided written informed consent. Children 6–17 y/o provided verbal assent.

### Study recruitment

All participants were recruited from the HCSFV during the 2014 and 2015 chikungunya epidemics in Managua. During enrollment, study personnel administered informed consent and recruited participants based on laboratory-confirmed or clinically/epidemiologically probable presentation of chikungunya. Individuals aged 6 months and older were considered for the study. Inclusion criteria for the study were as follows: 1) a laboratory-confirmed or clinically/epidemiologically probable presentation of chikungunya, 2) having been attended to at the HCSFV, 3) residing in the HCSFV catchment area during the study period, 4) having provided written informed consent themselves or through a legal representative if <18 y/o, and 5) having provided verbal assent if between 6 and 17 y/o [3,4]. All pediatric patients between 2–14 y/o were active participants in the PDCS [29], and all participants were recruited from the HCSFV upon fulfillment of inclusion criteria.

All participants were identified as chikungunya cases by study physicians. Diagnostic criteria used during the 2014–2015 chikungunya epidemics were based on clinical standards for evaluating suspected arboviral infections that circulate in Managua. These criteria consisted of the World Health Organization’s case definition for chikungunya cases [30]. Suspected chikungunya cases were assessed for 1) undifferentiated fever or 2) fever or feverishness and 2 or more of the following: headache, muscle pain, joint pain, retro-orbital pain, rash, hemorrhagic manifestations, and leukopenia [22]. Suspected cases were confirmed as CHIKV infections if 1) CHIKV real-time reverse transcriptase-polymerase chain reaction (rRT-PCR) results were positive 2) seroconversion was observed using an anti-CHIKV immunoglobulin M enzyme-linked immunosorbent assay (ELISA) using paired acute and convalescent samples and/or 3) seroconversion was detected by Inhibition ELISA in paired acute and convalescent samples [23]. Of suspected cases, 682 were confirmed as positive by rRT-PCR, and 53 were confirmed as positive by seroconversion.

Thirty-five participants were recruited beyond the acute phase after being diagnosed as clinically/epidemiologically probable chikungunya cases at the HCSFV; they were included in the long-term follow-up and analysis. Laboratory diagnosis was not conducted for these patients as they lacked acute-phase serum samples. Such participants were included in the analysis because they were judged by our study’s physicians to be clinically/epidemiologically probable chikungunya cases, as during the chikungunya outbreaks, there was very low transmission of other arboviral diseases.

Participants above the age of 55 were excluded from this study due to low sample sizes. Follow-up visit data were considered up to a maximum of 625 days post-fever onset, due to low sample sizes beyond day 625. Of the 811 participants eligible for the study, 41 were excluded due to disqualifying, missing, or incomplete data, bringing the size of the analytic population to 770 individuals.

### Parallel Zika cohort

Participants from a longitudinal pediatric cohort of laboratory-confirmed and symptomatic ZIKV infections (n = 161), recruited from the PDCS during the 2016 ZIKV epidemic after the CHIKV epidemics, followed similar inclusion criteria but with clinical and laboratory diagnostic methods for Zika [31]. These Zika cases were monitored for ~18 months for the occurrence of arthralgia. The Zika cohort study ran parallel to the chikungunya cohort study and involved the same study recruitment procedures and study design. Participants in the Zika cohort study were diagnosed based on established PDCS clinical criteria for suspected Zika illness, and all were confirmed via rRT-PCR [31]. Our comparison of the two cohorts was conducted with the consideration that Zika may manifest similarly to chikungunya in the acute phase of disease, but it has not been shown to contribute to chronic arthralgia [8,32]. Moreover, we have previously shown that among PDCS participants who report to the HCSFV and who meet our testing definition, the percentage of arthralgia among Zika and non-Zika cases is not significantly different (prevalence difference = 1.5%, p = 0.57)[31]. This suggests that Zika cases are a good comparison for understanding the baseline levels of pediatric arthralgia in our study setting.

### Study design

Participants were clinically evaluated upon enrollment and followed up at approximately 15 days and 1, 3, 6, 12, and 18 months post-fever onset. Follow-up evaluations were conducted at the participants’ residence (home visits) or at the medical center, depending on the specific circumstances. At each study follow-up visit, study medical professionals administered a questionnaire to the participant or legal guardian and then conducted a physical examination that queried for arthralgia (S1 Appendix) [31]. Arthralgia was defined as verbal or physical indications of joint pain, discomfort, or inflammation. The occurrence of arthralgia was assessed across the neck, shoulders, back, hips, elbows, wrists, hands, knees, ankles, and feet. The study period, covering both chikungunya epidemics and the follow-up visits, extended from September 2014 to January 2018. Pediatric participants 2–14 y/o were co-enrolled in the PDCS [29]. If participants demonstrated either persistent or apparent symptoms of disease, they were referred to seek further care at the medical center. Participants who required pharmacological treatment were recommended for non-steroidal anti-inflammatory drugs, mainly acetaminophen and ibuprofen. All participants requiring medical attention received care at the HCSFV throughout the study.

### Phases of chikungunya

Participants with chikungunya were classified as experiencing acute, interim, or chronic arthralgia. Acute cases were defined as experiencing illness strictly within the first 10 days post-fever onset based on CDC and World Health Organization (WHO) guidelines [7,13]. Acute chikungunya was initially stratified by both ≤15 days and ≤10 days post-fever onset in our analysis, with no meaningful difference in results. Chronic chikungunya cases were defined as experiencing chikungunya-associated signs or symptoms ≥90 days post-fever onset based on WHO and French guidelines from studies of outbreaks on Réunion Island [4,13]. The interim cases covered the period from 11–89 days post-fever onset [4,10]. Collectively, the interim and chronic cases constitute the *post-acute* phase of chikungunya, defined as any report of arthralgia >10 days post-fever onset. For our analysis, participants who experienced arthralgia in multiple chikungunya phases were analyzed by the last phase in which they reported arthralgia.

### Statistical methods and data analysis

Participants were stratified into four age ranges: 0–4, 5–9, 10–15, and 16+ y/o based on established PDCS protocols and the Nicaraguan Ministry of Health. Adults were grouped into one range, 16+, due to the small sample size that prohibited further stratification. When directly comparing pediatric and adult cases, the pediatric group was condensed to ages ≤15 and the adult group consisted of ages 16+.

We used logistic regression to estimate odds ratios (ORs) for binary outcomes, such as the presence or absence of arthralgia. We considered both unadjusted models and models adjusted for sex and age. In our analysis modeling the relationship between the three phases of disease (*i*.*e*., acute, interim, and chronic), we adjusted for age and sex based on supporting evidence from the literature and our own analyses [10]. Generalized additive models (GAM), semi-parametric extensions of generalized linear models (GLM), were used to visualize the non-linear relationship between different variables such as age, chikungunya-associated arthralgia across different parts of the body, and phases of chikungunya disease [33]. GAMs were preferred over GLMs due to the addition of smoothing functions that better estimate the functional relationships between the outcome and associated explanatory variables. GAMs relax GLMs’ assumption of linearity, allowing for the estimation of non-linear trends [33].

Pearson’s Chi-squared test was used to compare the proportion of participants with arthralgia across age groups and sex [34]. Survival analyses were used to describe the percentage of participants reporting arthralgia over time, accounting for right censoring and stratified by age, sex, and diagnosis. Survival data were visualized using Kaplan-Meier (KM) curves, which plot the probability of an outcome not occurring across the study period [35]. KM survival curves were evaluated using the log-rank test, with the null hypothesis proposing that the plotted survival curves are similar. We evaluated group trends in the KM survival curves using the log rank test for trend [36]. We omitted confidence intervals in the visualization of the KM survival curves to improve graphical representation of the data, and we used the p-value from the log-rank test to quantify statistical differences between group-specific survival curves. Weibull survival models were used to calculate the hazard ratio (HR) of experiencing the outcome (presence or absence of arthralgia) over the study period, compared to a reference group [36]. Survival analyses were utilized only when observing the data beginning from the interim phase of disease (>10 days post-fever onset) until study cessation, as the proportional hazard assumption was not satisfied during the acute phase. Survival analyses were conducted using the survival, survminer, and eha R packages and visualized in base R [36–38].

Agglomerative hierarchical clustering was used to construct a co-occurrence dendrogram of participants’ signs and symptoms [39]. The Ward method was used to create groups with minimal variance, and the Manhattan distance was used to determine the underlying distance matrix. The cophenetic distance correlation coefficient was calculated to measure the similarity between the original distance and the cophenetic distance [39]. The higher the cophenetic distance correlation coefficient is the more appropriately the dendrogram represents a hierarchical structure present in the original data. Analyses were conducted using the base stats package, and dendrograms were visualized using base R graphics and the dendextend package [40].

For a sensitivity analysis, we used KM estimates to evaluate any differences in reported arthralgia occurrence between the rRT-PCR-confirmed and clinically/epidemiologically probable groups. Further, to evaluate whether baseline arthralgia among pediatric individuals might confound our results, we conducted a comparison between our pediatric chikungunya cases and the pediatric Zika cases. We compared the reported arthralgia occurrence beyond the acute phase of disease using KM estimates.

Data were analyzed using R version 4.1.1 within the RStudio (2021.09.0, Build 351) integrated development environment [41]. Data management was conducted using the core tidyverse packages along with base R, lubridate, reshape2, broom, epitools, plyr, and tableone packages [41–47]. Two-dimensional plots were visualized using base R graphics and the ggplot2 R package, supplemented with the scales package to express percentages [48,49]. Tables were created using Microsoft Word 2021 (v16.56).

## Results

### Participant characteristics

Participants consisted of 770 chikungunya cases; 682 (88.6%) were positive by real-time RT-PCR, and 88 (11.4%) were clinically/epidemiologically probable cases. Study participants’ median age was 11 y/o (interquartile range: 7–14) (S1 Fig). We enrolled 394 (51.2%) females and 376 (48.8%) males. There were 612 pediatric (0–15 y/o) and 158 adults (16+ y/o) cases (Table 1). During the chikungunya epidemics, there were very few cases of dengue – which can present similarly as chikungunya – detected in the PDCS, limiting the number of incorrectly classified chikungunya cases based on clinical/epidemiological criteria.

**Table 1:** Characteristics of the participants in the chikungunya prospective cohort study in Managua, Nicaragua (2014–2018).

Variable	Data (n = 770) ^a^
**Age, median [IQR]**	
Age (years)	11 [7.0, 14.0]
**Sex, n (%)**	
Female	394 (51.2)
Male	376 (48.8)
**Confirmation method, n (%)**	
Real-time RT-PCR-positive	682 (88.6)
Serologically confirmed or clinically/epidemiologically probable	88 (11.4)
**Epidemic, n (%)**	
Epidemic 1 (2014–15)	207 (26.9)
Epidemic 2 (2015–2016)	563 (73.1)
**Age range (years), n (%)**	
0–4	105 (13.6)
5–9	200 (26.0)
10–15	307 (39.9)
16+	158 (20.5)
16–25	71 (44.9)
26–35	38 (24.1)
36–45	32 (20.3)
46–55	17 (10.9)

^a^ Visits after day 625 and participants >55 years of age were excluded.

### Age-based differences in chikungunya-associated arthralgia

**Fig 1:**
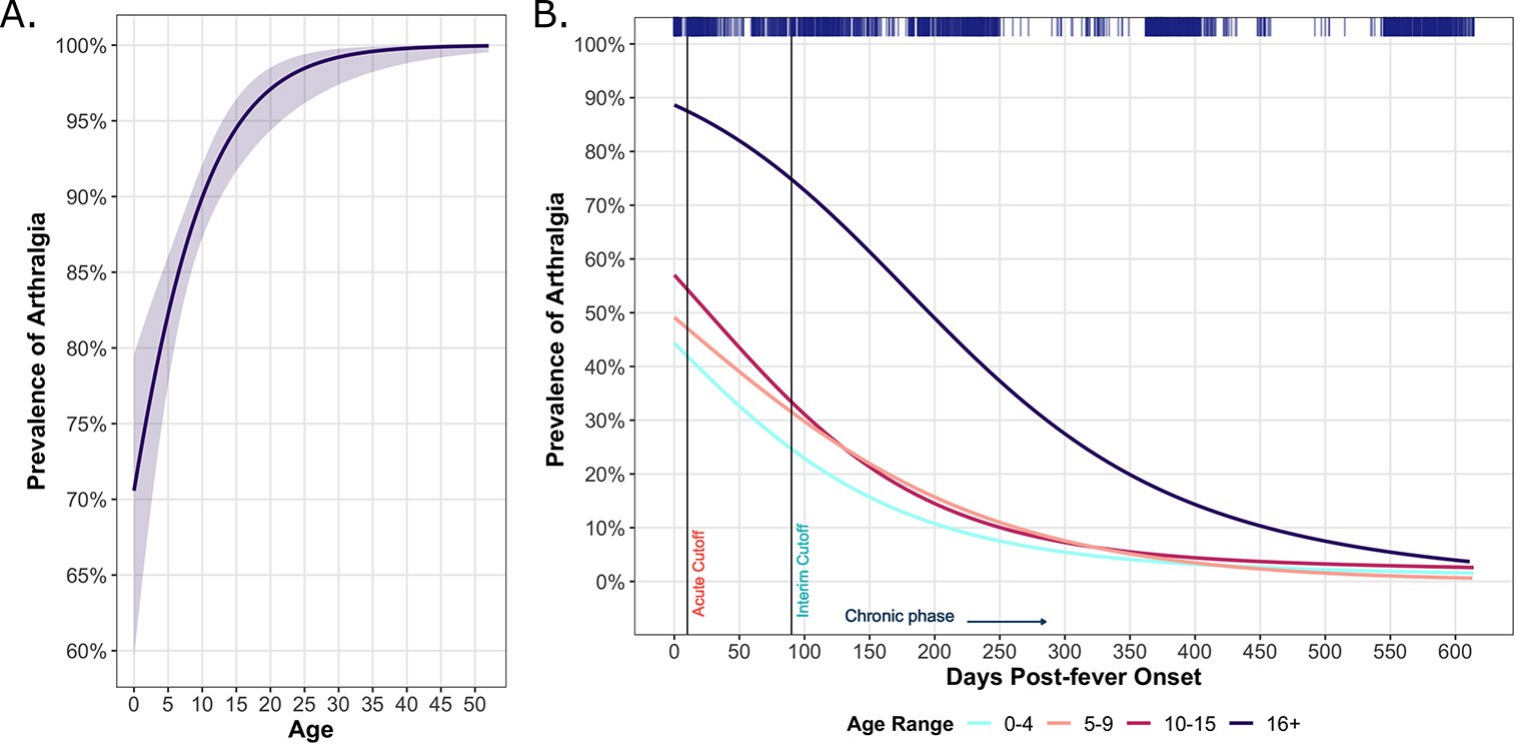
Prevalence of post-chikungunya-associated arthralgia over time, stratified by age range in years in Managua, Nicaragua (2014–2018). Age trends for the prevalence of post-chikungunya-associated arthralgia depicted using a generalized additive model. A 95% confidence band is shown around the mean trend (A). The prevalence of arthralgia measured across days since fever onset and stratified by age range is depicted using a generalized additive model. Distributed marks at the top indicate the density of patient responses by day since fever onset. Participants were considered as having either acute (<10 days), interim (>10 and <90 days), or chronic (>90 days) disease (B).

**Fig 2:**
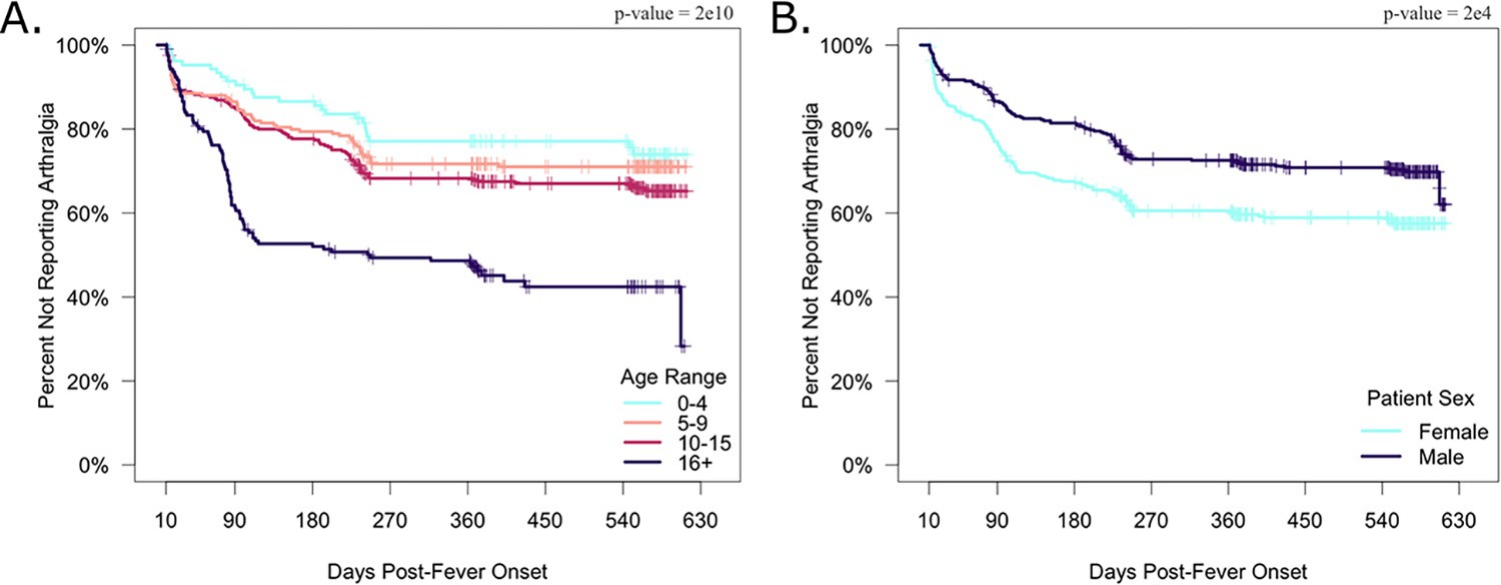
Kaplan-Meier plot showing the proportion of participants reporting arthralgia over time in years in Managua, Nicaragua (2014–2018). A Kaplan-Meier graph plotting the proportion of participants not reporting arthralgia (y-axis) against days since fever onset (x-axis). Ticks correspond to censoring events. Panels show the distribution of participants beginning 10 days post-fever onset and ending at the last reported data point based on the exclusion criteria (< 625 days post-fever onset), stratified by age range (A) and sex (B). The p-values were calculated using the log-rank test.

Overall, 88% of pediatric and 98% of adult participants reported arthralgia during the study period (Table 2). We observed that the prevalence of arthralgia increased in an age-dependent manner (Fig 1A) but decreased over time since fever onset for all age groups (Fig 1B). KM plots demonstrated an age-dependent increase in the percentage of participants who reported post-acute arthralgia across ~1.5 years of follow-up time (Fig 2A). The pediatric groups reported ~30% of their total instances of arthralgia occurring beyond the acute phase, showing the substantial burden of chikungunya-associated arthralgia among children (Table 3). Crucially, a large percentage of pediatric participants (0–4 y/o: 18.6%; 5–9 y/o: 23.2%; 10–14 y/o: 32.5%) reported 2+ visits with symptoms of arthralgia (Table 4). The adult group reported 43.4% of their total instances of arthralgia in the post-acute phase, a significantly higher proportion compared to all other age groups (p-value < 0.05) (Table 3). Adults reported the highest percentage (55.1%) of individuals with 2+ visits with symptoms of arthralgia (Table 4). Age-dependent trends in reported arthralgia occurrence were supported by KM estimates (Fig 2A), ORs (Table 2), and HRs (S1 Table).

**Table 2:** Risk factors for acute chikungunya-associated arthralgia in Managua, Nicaragua (2014–2018).

Category	Reported Arthralgia		Odds Ratio (95% CI ^a^)
	No; n (%)	Yes; n (%)	
**Age range (years)**			
0-4	23 (22.6)	79 (77.5)	0.27 (0.14, 0.51)
5-9	30 (15.2)	168 (84.8)	0.44 (0.24, 0.78)
10-15	22 (7.3)	280 (92.7)	-
16+	2 (1.6)	125 (98.4)	4.91 (1.42, 30.95)
**Sex**			
Female	31 (8.3)	341 (91.7)	1.63 (1.01, 2.65)
Male	46 (12.9)	311 (87.1)	-
**Total** ^**b**^			
All participants	77 (10.6)	652 (89.4)	-

^a^ CI = Confidence Interval

^b^ Some participants were omitted due to missing acute-phase data, n = 41

**Table 3:** Prevalence of reports of arthralgia in the acute and post-acute phase in Managua, Nicaragua (2014–2018).

Age range	Acute, n (%)	Post-acute, n (%)	Total ^a^
0-4	73 (73.7)	26 (26.3)	99
5-9	158 (67.8)	75 (32.2)	233
10-15	275 (68.8)	125 (31.3)	400
16+	124 (56.6)	95 (43.4)	219

^a^ Some participants were omitted due to missing acute data, n = 41

**Table 4:** Proportion of visits with reports of arthralgia in Managua, Nicaragua (2014–2018).

Age range	Number of visits with reported arthralgia ^a^		
	0, n (%)	1, n (%)	2+, n (%)
0-4	23 (22.5)	60 (58.8)	19 (18.6)
5-9	30 (15.2)	122 (61.6)	46 (23.2)
10-15	22 (8.3)	182 (60.3)	98 (32.5)
16+	2 (1.6)	55 (43.7)	70 (55.1)

^a^ Some participants were omitted due to missing acute data, n = 41

### Clinical presentation of post-acute chikungunya-associated polyarthralgia

**Fig 3:**
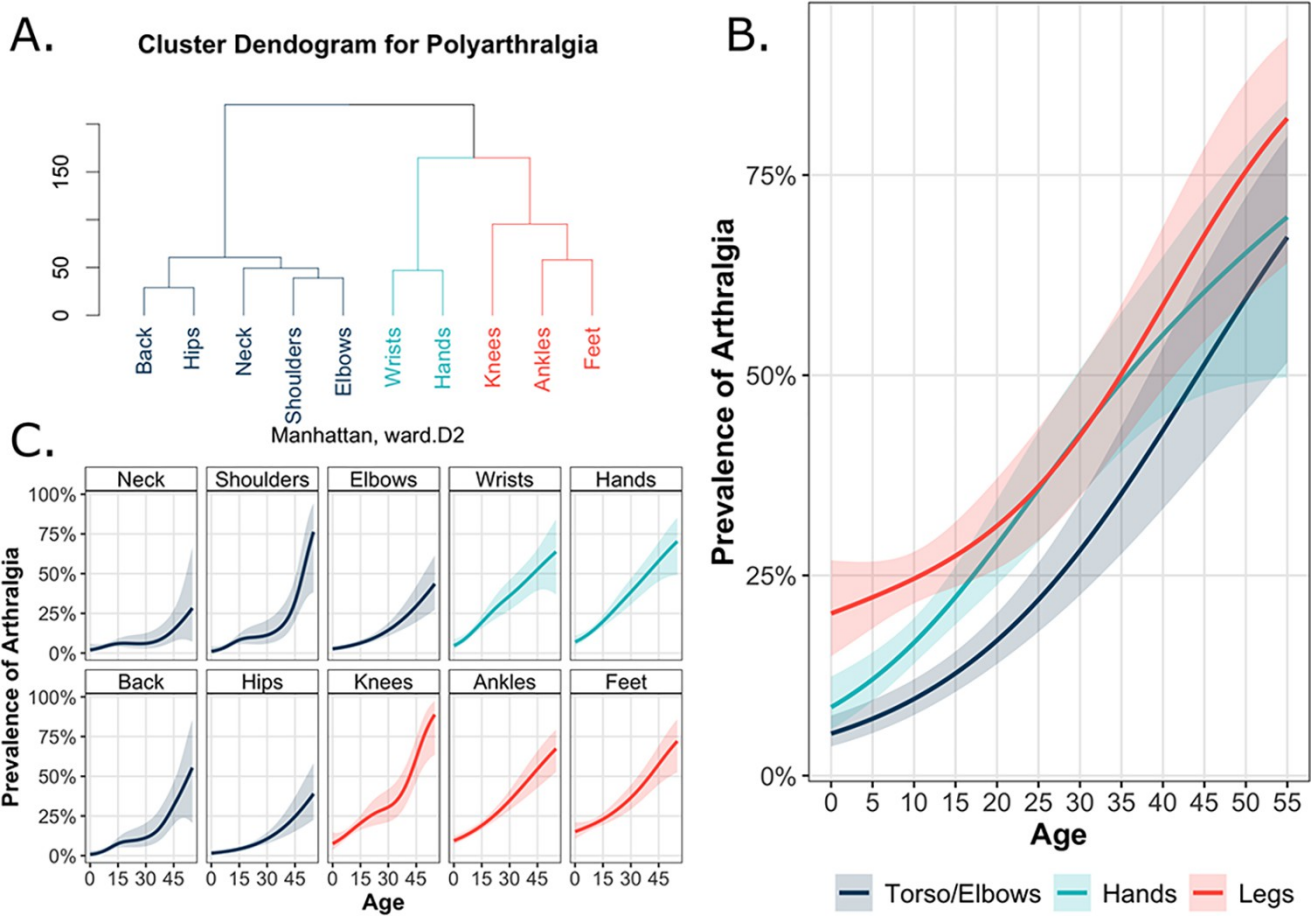
Reported polyarthralgia beyond the acute phase of chikungunya associated-arthralgia by body part and age in year in Managua, Nicaragua (2014–2018). Cluster dendrogram depicting the relationship between occurrence of polyarthralgia across the different body parts, with the y-axis representing the underlying cluster distance calculated using the Manhattan distance method. The cophenetic distance correlation coefficient is 0.95; the higher the cophenetic distance correlation coefficient is, the more appropriately the dendrogram represents a hierarchical structure present in the original data (A). Age trends of the prevalence of arthralgia among clustered body groups (B) and individual body parts (C), including the 95% confidence intervals, visualized using shading corresponding to each respective color group and depicted using a generalized additive model.

Clustering analyses demonstrated that polyarthralgia co-occurred in three general areas, constituting distinct clusters of localized arthralgia: the legs (knees, ankles, and feet), hands (wrists and hands), and torso/elbows (neck, shoulders, back, hips, and elbows) (Fig 3A). The most fundamental difference in polyarthralgia co-occurrence was the division of the hand and leg areas, which clustered together, from the torso/elbows, which formed its own cluster.

The prevalence of arthralgia increased across age for each of the three clusters identified through hierarchical clustering (Fig 3B). The prevalence of polyarthralgia was highest in the legs (28.5%), followed by the hands (21.1%) and the torso/elbows (13.5%) (S2 Table). The proportion of polyarthralgia between distinct body parts was significantly different when combining all age groups (S2 Table). Specifically, among pediatric participants (≤15 y/o), the difference between localized polyarthralgia in the hands and legs was significantly different (p-value < 0.01), while this difference was not observed in adults (>15 y/o, p-value = 0.43), indicating distinct presentation of polyarthralgia between the two groups. For each cluster, the prevalence of polyarthralgia increased substantially and linearly across age; linearity is particularly evident after age 20 (Fig 3B). Such trends represent averages across the underlying body parts, which we also characterized. In general, the age-specific prevalence of arthralgia for the individual body parts resembled the trends of the corresponding cluster (Fig 3C), supporting the clustering analysis and extending its results across age.

### Sex-based differences in chikungunya-associated arthralgia

Over 85% of both males and females reported arthralgia over the study period. Over 18 months of follow-up, females experienced significantly higher odds of arthralgia (OR: 1.63 [95% CI: 1.01–2.65]) than males (Table 2). Beyond the acute phase of disease, a significantly higher proportion of females experienced arthralgia compared to males (p < 0.001; log rank test) (Fig 2B). During this same time period, the hazard for females experiencing arthralgia was also significantly higher compared to males (HR: 2.27 [95% CI: 1.49–3.46]) (S1 Table), both among children (HR: 1.97 [95% CI: 1.18–3.28]) and adults (HR: 2.20 [95% CI: 1.11–4.35]). Sex-based differences in arthralgia occurrence were most apparent within the first six months of follow-up, and this difference remained relatively unchanged until study cessation (Fig 2B). Altogether, these data suggest distinct, sex-based experiences of arthralgia that are especially pronounced beyond the acute phase.

### Differences across the acute, interim, and chronic phases

**Fig 4:**
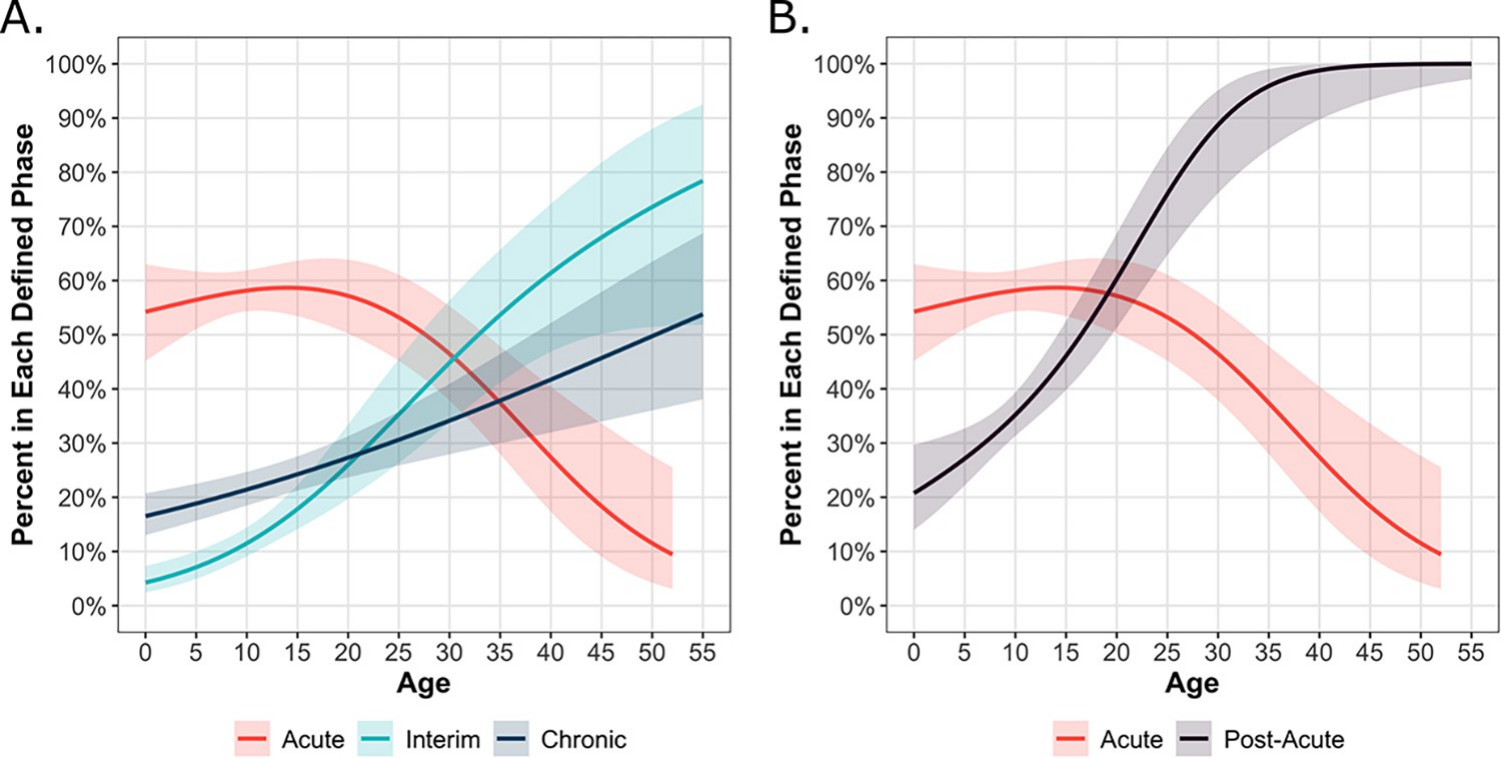
Age trends in years for the percentage of chikungunya-associated arthralgia cases in each defined phase in Managua, Nicaragua (2014–2018). Participants were considered as acute (<10 days), interim (>10 days and <90 days), or chronic (>90 days) phase arthralgia cases (A) or considered as either acute (<10 days) or post-acute (>10 days) phase arthralgia cases (B), based on their last instance of arthralgia. Graphs include the 95% confidence intervals, visualized using shading corresponding to each respective color group and depicted using a generalized additive model. The y-axis reflects, out of all participants with reported arthralgia, what percent had their last instance of arthralgia in each given phase.

We then evaluated the proportion of acute, interim, and chronic arthralgia cases, defined by the last instance of reported arthralgia, across continuous age. We observed that as age increased, the proportion of acute cases decreased while the proportion of interim or chronic cases increased (Fig 4A). Among younger participants (<15 y/o), ~20% were considered chronic and ~10% interim cases, with ~55% considered acute cases and a subset reporting no arthralgia (~15%) (S3 Table). A high proportion of the older participants were considered interim (26.0%) and chronic cases (29.1%), while fewer were defined as acute cases (43.3%) or did not report arthralgia (1.6%) (S3 Table). At ~18 years of age, the proportion that experienced interim or chronic arthralgia increased up to 50%, with the percentage increasing dramatically as age increased (Fig 4A). Indeed, by age 30, the proportion of chronic and interim cases was approximately 90%. When comparing pediatric (<15 y/o) and adult (>15 y/o) participants, we observed that adults had a significantly higher proportion of interim cases (p-value < 0.01) but not chronic cases (p-value = 0.06). This difference is driven by the significantly lower proportion of post-acute pediatric cases being defined as interim cases (29.4%) rather than chronic cases (70.6%, p-value < 0.01), a difference not observed between interim (47.1%) and chronic (52.9%) cases among adults (p-value = 0.67). Thus, differences in the presentation of arthralgia between the pediatric and adult groups were primarily driven by the occurrence of arthralgia during the interim period.

### Association between the interim and chronic phases

The age-specific trends of interim and chronic arthralgia cases were broadly similar to each other in children, but the trends appeared to differ more among adults (Fig 4A and S3 Table). To examine this more thoroughly, we used logistic regression to quantify the association between having either acute or interim arthralgia (the exposure variables) and later developing chronic arthralgia (the outcome variable). Among pediatric (0–15 y/o) participants, having interim arthralgia was significantly associated with exhibiting chronic arthralgia, though there was no evidence that experiencing acute arthralgia was associated with developing chronic arthralgia (Table 5). Similar results were obtained after adjusting for sex and continuous age. However, among adults, the association between interim arthralgia and chronic arthralgia was not statistically significant in either unadjusted or adjusted models.

**Table 5:** Odds of progressing to chronic chikungunya-associated arthralgia after an initial presentation with only acute or interim chikungunya-associated arthralgia in Managua, Nicaragua (2014–2018).

Category	Odds ratio (95% CI ^a^)	
	Unadjusted	Adjusted ^b^
**All ages**		
Acute arthralgia ^c^	1.65 (0.96, 3.01)	1.36 (0.78, 2.50)
Interim arthralgia	2.31 (1.50, 3.55)	1.95 (1.19, 3.14)
**0-15 y/o**		
Acute arthralgia	1.44 (0.83, 2.65)	1.27 (0.72, 2.36)
Interim arthralgia	2.12 (1.24, 3.57)	2.06 (1.20, 3.47)
**16+ y/o**		
Acute arthralgia	0.92 (-1.62, 5.86)	1.08 (-1.57, 6.04)
Interim arthralgia	0.14 (-0.99, 1.31)	0.50 (-0.78, 1.87)

^a^ CI = Confidence Interval

^b^ Adjusted for age and sex

^c^ Participants were considered as having either acute (≤10 days), interim (>10 and <90 days), or chronic (≥90 days) disease

As the regression results strengthened our finding that the interim and chronic phases were only similar in the pediatric group, we analyzed the proportion of acute and post-acute (combining the interim and chronic cases) arthralgia cases over continuous age (Fig 4B). The proportion of post-acute cases increased linearly with continuous age, ranging from 25% to 45% among the youngest ages (0–15 y/o) and overtaking the proportion of acute cases around age 20 y/o before reaching ~100% by the age of 35 y/o (Fig 4B). Further, we observed a significant difference between the proportion of pediatric and adult patients with post-acute arthralgia (p-value < 0.01).

In sum, our analysis suggests that a child experiencing interim arthralgia is also likely to experience chronic arthralgia; however, having interim arthralgia as an adult is not associated with chronic pain.

### Sensitivity analysis

**Fig 5:**
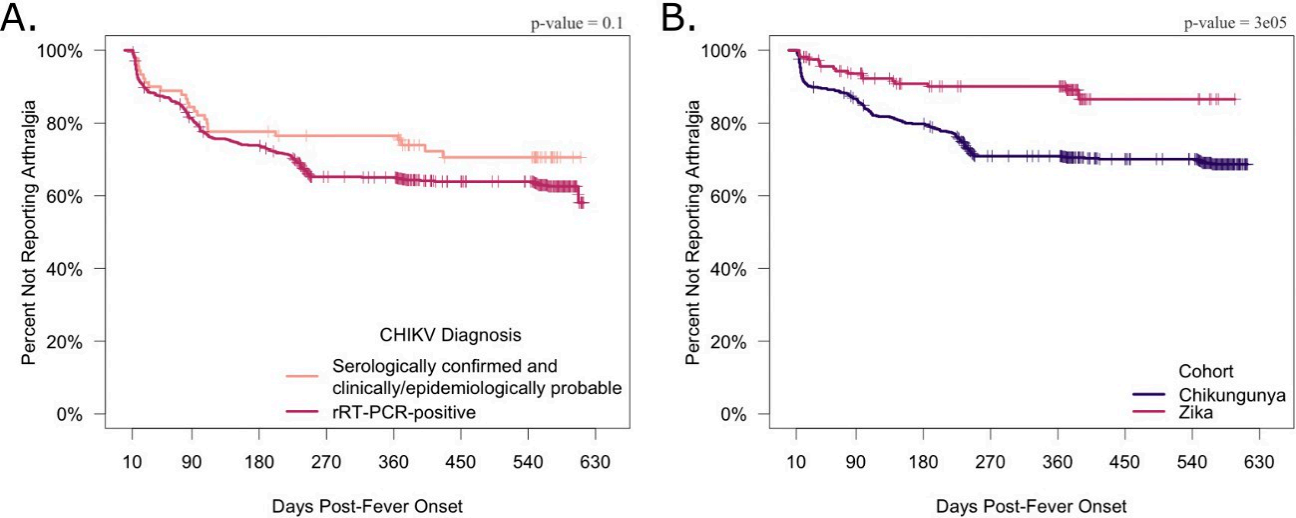
Kaplan-Meier plot demonstrating the proportion of participants not experiencing arthralgia by diagnostic method and cohort. A Kaplan-Meier graph plotting the proportion of participants not reporting arthralgia (y-axis) against days since fever onset (x-axis). Ticks correspond to censoring events. Panels show the distribution of participants beginning 10 days post-fever onset and ending at the last reported data point based on the exclusion criteria (<625 days post-fever onset), stratified by cohort (A) or CHIKV infection diagnostic method (B). The p-values were calculated using the log-rank test.

As a final analysis, we examined whether the confirmation method and baseline levels of pediatric arthralgia impacted our major results. Real-time RT-PCR-confirmed participants and serologically confirmed and clinically/epidemiologically probable participants showed no differences in reported arthralgia occurrence over time (Fig 5A). However, the chikungunya cohort reported a significantly higher percentage of arthralgia occurrence beyond the acute phase of disease compared to the parallel Zika pediatric cohort, consistent with known clinical differences between chikungunya and Zika. This demonstrates that the levels of arthralgia observed in the cohort were distinct from baseline pediatric arthralgia (Fig 5B).

## Discussion

We describe risk factors and characteristics of long-term arthralgia among 770 chikungunya cases in a prospective cohort in Managua, Nicaragua. We find that pediatric chikungunya cases are most vulnerable to chikungunya-associated arthralgia during the acute phase, but they do exhibit a meaningfully high prevalence of arthralgia occurring beyond the acute phase of disease. Further, we demonstrate an age-dependent increase in the prevalence of arthralgia from infancy throughout adulthood. While the association between age and arthralgia has been described in adults [18], we present one of the first analysis of longitudinal pediatric chikungunya-associated arthralgia [15]. The highest prevalence of post-acute pediatric polyarthralgia was reported in the legs, followed by the hands and torso/elbows; no significant differences among body parts was observed in adults, though the sample size of adults was limited. Finally, when comparing the three phases of chikungunya described in literature, we observed a strong similarity between interim and chronic arthralgia phases in pediatric cases (≤15 y/o), but not in adult cases (>15 y/o). Our findings provide new insights into chikungunya-associated arthralgia in both pediatric and adult cases that could be used to improve clinical guidelines.

There are limited longitudinal data on the age-specific changes in the risk of chronic arthralgia due to chikungunya, particularly in Latin American populations. Our regression model demonstrated that a substantial ~77.5–92.7% of pediatric cases reported arthralgia, and of the total arthralgia reported among pediatric cases, ~26.3–31.3% occurred beyond the acute phase, demonstrating that many pediatric cases are vulnerable to long-term arthralgia. Further, we observed that the proportion of acute, interim, and chronic cases were approximately equal around age 30–35, whereas the transitional age decreased to approximately 20 years when the interim and chronic phases were considered together. Notably, age 20 is far below the threshold of 45 years commonly used in clinical guidelines to demarcate risk for post-acute arthralgia in chikungunya cases. Thus, an opportunity exists for guidelines [4,10,17] to decrease the threshold age to capture more patients, thereby leading to improved case management and patient outcomes. These observations highlight the burden of chronic, chikungunya-associated arthralgia beginning at a younger age than often described in the literature [10,17,18]. Importantly, although quality-of-life research in children with chikungunya is lacking, several studies have demonstrated the negative effect of arthralgia on the livelihood of adult cases [50,51]. In a study conducted on La Réunion Island in 2012, adult cases reported arthralgia, discomfort, and depression up to six years after acute chikungunya, and 48% of the chikungunya patients declared moderate to intense pain compared to 16% of non-chikungunya cases [9]. If clinical guidelines do not fully account for the hidden burden of chikungunya in children and young adults, these groups will be at heightened risk of being neglected, might not receive appropriate medical management, and will suffer negative effects on their activity and education, particularly in resource-limited settings [14,15].

We observed that polyarthralgia in the legs was most pronounced in children. Thus, examination of the legs may serve to diagnose post-acute arthralgia better than other body parts in children. Further, we note that arthralgia in the elbow clusters with arthralgia in the neck, back, shoulders, and hips, despite the proximity of elbows to the hands and wrists. Little is known about how arthralgia in the joints clusters and manifests post-acutely in children [10,15]. Understanding the unique presentation of polyarthralgia among different age groups can facilitate identification of chikungunya, particularly among young children with a limited capacity to detail their pain. The relatively high prevalence of post-acute polyarthralgia suggests long-term follow-up should be conducted for both children and adults.

Our results extend the observed trend in the literature that older females (50+ y/o) experience arthralgia more often than older males [18,52] to children, as we found that pediatric females (0–15 y/o) had significantly higher odds and post-acute hazards of arthralgia when compared to males. Hormonal and immunological differences between females and males has been hypothesized [53] to explain the higher prevalence of arthritic diseases among females, though similarities between chikungunya-associated arthralgia and rheumatoid arthritis are debated [11,54]. Altogether, our results and the literature suggest that across all ages, sex is an important risk factor for arthralgia. Consequently, it is critical for clinical guidelines to emphasize the risk and management of chikungunya-associated arthralgia for females of any age.

The WHO recommends that the chronic phase of chikungunya be defined as 12 weeks post-symptom onset [13], with little acknowledgment of the interim phase. Here, we observed distinct trends and presentation of arthralgia in pediatric participants between the first 10 days post-fever onset (acute phase) and thereafter (interim and chronic phases). To our knowledge, no analysis to date has described the prevalence of arthralgia in each phase of chikungunya from childhood to adulthood. Furthermore, the divergence between the acute and interim/chronic phases we observed was particularly evident between age groups. This finding suggests that although interim-phase arthralgia in adults is self-limiting, interim-phase arthralgia in children differs from adults and can be utilized as an indicator for chronic arthralgia. Vairo et al. [16] and the French Infectious Diseases Society [4] have previously highlighted the similarities between the interim and chronic phases. It has been noted that earlier and more successful clearance of CHIKV during the acute phase results in protection from chronic chikungunya [55,56]. Further, early management of acute inflammation (by use of steroidal and non-steroidal anti-inflammatory drugs) has been shown to decrease the risk of chronic inflammation [57]. Interim arthralgia might be linked to poor viral clearance and constitutes a higher risk for long-term symptoms. If guidelines explicitly associate pediatric interim arthralgia with greater odds of chronic arthralgia, medical professionals could identify cases with earlier signs of arthralgia as being at risk for prolonged arthralgia and ensure long-term follow-up to best manage pediatric cases.

Our study has several limitations. Our results are based in part on reports of polyarthralgia from pediatric patients, which might introduce misclassification bias due to subjectivity when clinically probing for signs of arthralgia. However, our data is collected by physicians who individually have over 10 years’ of experience in diagnosing arthralgia and related conditions in young children due to the high burden of arboviral disease in Managua [31]. Further, our study is limited by the small sample size of adult participants, which was by design as we aimed to primarily characterize children so as to fill gaps in the literature. We were unable to conduct comprehensive analyses on the severity of arthralgia and changes in pain across body parts over time due to limitations in our study questionnaire. Finally, the generalizability of our findings may be limited to the Asian CHIKV lineage, due to the previously described variability in clinical presentations of chikungunya across CHIKV lineages [18,24].

Overall, our results provide new insights into chikungunya-associated arthralgia and demonstrate the high prevalence of arthralgia in pediatric cases, both in the acute and post-acute phases. We observe a strong age-prevalence trend for arthralgia and suggest improvements for the pediatric definition of the phases of chikungunya. Overall, our results inform chikungunya clinical guidelines for short-term and long-term care in both pediatric and adult populations.

## Supporting information

S1 Table Hazards for chikungunya-associated arthralgia >10 days post-fever onset in Managua, Nicaragua (2014–2018). (DOCX)

S2 Table Prevalence of post-acute polyarthralgia by age and body part in Managua, Nicaragua (2014–2018). (DOCX)

S3 Table Prevalence of each phase of chikungunya-associated arthralgia by age in Managua, Nicaragua (2014–2018). (DOCX)

S1 Fig Density plot of participants stratified by age range in years in Managua, Nicaragua (2014–2018). The percent of participants in this study across age are depicted using an age-density plot. Colors correspond to the age-ranges defined within the study (0–4, 5–9, 10–15, and 16+ years old).
(TIF)

S1 Appendix Questionnaire for the Retrospective and Prospective Study of Clinical, Virological, and Immunological Characteristics of Chikungunya Cases in Nicaragua. (DOCX)
