# Supplementary material for: Longitudinal analysis of post-acute chikungunya-associated arthralgia in children and adults: A prospective cohort study in Managua, Nicaragua (2014–2018)
Source: PLoS Negl Trop Dis. 2024 Feb 28;18(2):e0011948. doi: 10.1371/journal.pntd.0011948 (PMC10962812; doi:10.1371/journal.pntd.0011948)
Supplement: S1 Table — (DOCX) [file pntd.0011948.s001.docx]

**S1 Table. Hazards for chikungunya-associated arthralgia >10 days post-fever onset in Managua, Nicaragua (2014-2018).**

| **Category** | **Hazard ratio (95% CI*)** |
| --- | --- |
| **Age range** | |
| 0-4 | 0.51 (0.25, 1.07) |
| 5-9 | 0.74 (0.43, 1.27) |
| 10-15 | - |
| 16+ | 3.72 (2.28, 6.05) |
| **Sex** | |
| Female | 2.27 (1.49, 3.46) |
| Male | - |
| *CI = Confidence Interval | |
