# Supplementary material for: Longitudinal analysis of post-acute chikungunya-associated arthralgia in children and adults: A prospective cohort study in Managua, Nicaragua (2014–2018)
Source: PLoS Negl Trop Dis. 2024 Feb 28;18(2):e0011948. doi: 10.1371/journal.pntd.0011948 (PMC10962812; doi:10.1371/journal.pntd.0011948)
Supplement: S2 Table — (DOCX) [file pntd.0011948.s002.docx]

**Supplemental Table 2. Prevalence of post-acute polyarthralgia by age and body part in Managua, Nicaragua (2014-2018).**

| **Category** | **Reported Arthralgia** | |
| --- | --- | --- |
|  | **No (%)** | **Yes (%)** |
| **All ages ^a^** | | |
| Legs | 549 (71.5) | 219 (28.5) |
| Hands | 605 (78.8) | 163 (21.2) |
| Torso | 664 (86.5) | 104 (13.5) |
| **Pediatric (< 15) ^a^** | | |
| Legs | 467 (76.3) | 145 (23.7) |
| Hands | 515 (84.1) | 97 (15.9) |
| Torso | 551 (90.0) | 61 (10.0) |
| **Adult (16+)** | | |
| Legs | 84 (52.9) | 73 (47.1) |
| Hands | 91 (58.0) | 66 (42.0) |
| Torso | 114 (72.6) | 43 (27.4) |
| ^a^ Comparisons of the equivalence of the prevalence of post-acute polyarthralgia in the legs, hands, and torso within each category are significantly different, p-value < 0.001 | | |
