# Supplementary material for: Longitudinal analysis of post-acute chikungunya-associated arthralgia in children and adults: A prospective cohort study in Managua, Nicaragua (2014–2018)
Source: PLoS Negl Trop Dis. 2024 Feb 28;18(2):e0011948. doi: 10.1371/journal.pntd.0011948 (PMC10962812; doi:10.1371/journal.pntd.0011948)
Supplement: S3 Table — (DOCX) [file pntd.0011948.s003.docx]

**Supplemental Table 3. Prevalence of each phase of chikungunya-associated arthralgia by age in Managua, Nicaragua (2014-2018).**

| **Age range** | **Acute (%) ^a^** | **Interim (%)** | **Chronic (%)** | **No Pain (%)** |
| --- | --- | --- | --- | --- |
| 0-4 | 55 (53.9) | 7 (6.9) | 17 (16.7) | 23 (22.5) |
| 5-9 | 113 (57.1) | 16 (8.1) | 39 (19.7) | 30 (15.2) |
| 10-15 | 179 (59.3) | 30 (9.9) | 71 (23.5) | 22 (7.3) |
| 16+ | 55 (43.3) | 33 (26.0) | 37 (29.1) | 2 (1.6) |
| ^a^ 41 participants were omitted due to missing acute-phase data | | | | |
