# Supplementary figures and images for: Longitudinal analysis of post-acute chikungunya-associated arthralgia in children and adults: A prospective cohort study in Managua, Nicaragua (2014–2018)
Source: PLoS Negl Trop Dis. 2024 Feb 28;18(2):e0011948. doi: 10.1371/journal.pntd.0011948 (PMC10962812; doi:10.1371/journal.pntd.0011948)

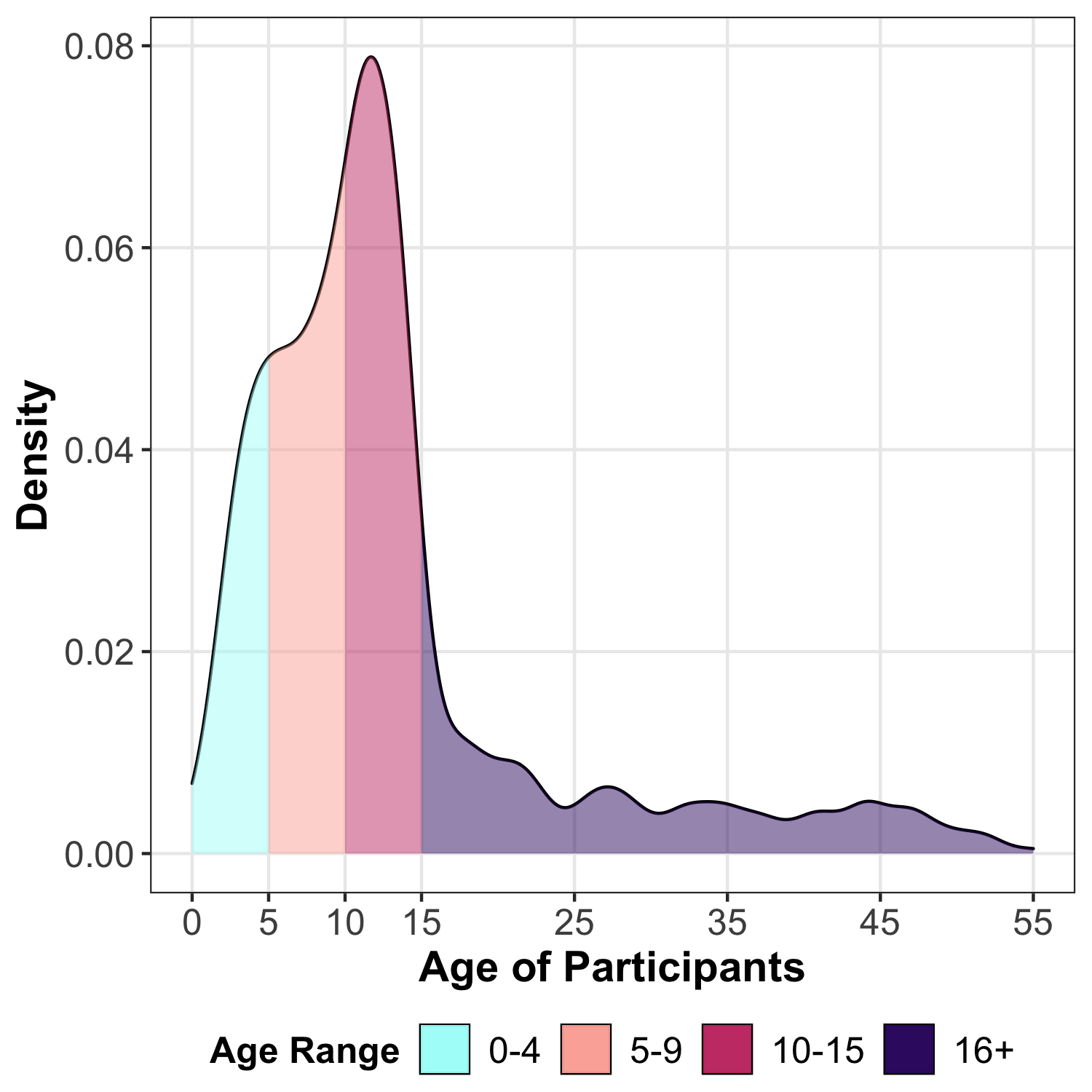

Supplement: S1 Fig — The percent of participants in this study across age are depicted using an age-density plot. Colors correspond to the age-ranges defined within the study (0–4, 5–9, 10–15, and 16+ years old). (TIF) [file pntd.0011948.s004.tif]
