# Supplementary material for: Longitudinal analysis of post-acute chikungunya-associated arthralgia in children and adults: A prospective cohort study in Managua, Nicaragua (2014–2018)
Source: PLoS Negl Trop Dis. 2024 Feb 28;18(2):e0011948. doi: 10.1371/journal.pntd.0011948 (PMC10962812; doi:10.1371/journal.pntd.0011948)
Supplement: S1 Appendix — (DOCX) [file pntd.0011948.s005.docx]

# Appendix

# Appendix 1. Questionnaire for the Retrospective and Prospective Study of Clinical, Virological, and Immunological Characteristics of Chikungunya Cases in Nicaragua

Estudio retrospectivo y prospectivo del comportamiento clínico, virológico, e inmunológico de casos de chikungunya en Nicaragua.

| Código del Participante: ____________ | Tipo de Contacto con el Participante:  Visita domiciliar  Vía Telefónica |
| --- | --- |
| Fecha de Visita: _____________ | Unidad de salud |
| Periodo de Visita: | Código del Encuestador |
| 15 Días |  |
| 1 Mes |  |
| 3 Meses |  |
| 12 Meses |  |
| 18 Meses |  |

Pregunta 1. Ud. o su niño aun continúan con dolor en una o más articulaciones?

Si______ No______

Si la respuesta es negativa no se realizara el resto de las preguntas

Si la respuesta es afirmativa, realizar la siguiente pregunta: Cual de las siguientes articulaciones presenta dolor?

|  | Si | No | Desconocido |
| --- | --- | --- | --- |
| Cuello |  |  |  |
| Hombros |  |  |  |
| Codos |  |  |  |
| Muñecas |  |  |  |
| Mano |  |  |  |
| Region Lumbar |  |  |  |
| Cadera |  |  |  |
| Rodillas |  |  |  |
| Tobillos |  |  |  |
| Pies |  |  |  |

Pregunta 2. La intensidad del dolor que Ud. o su niño perciben, lo puede valorar cómo?

|  | Nada | Poco | Bastante | Mucho |
| --- | --- | --- | --- | --- |
| Cuello |  |  |  |  |
| Hombros |  |  |  |  |
| Codos |  |  |  |  |
| Muñecas |  |  |  |  |
| Manos |  |  |  |  |
| Región Lumbar |  |  |  |  |
| Cadera |  |  |  |  |
| Rodillas |  |  |  |  |
| Tobillos |  |  |  |  |
| Pies |  |  |  |  |

Pregunta 3. El dolor que ud o su niño percibe le imposibilita realizar una o más actividades en su hogar?

Si_____ No_____

Si la respuesta es Afirmativa:

Qué Actividad le imposibilita realizar?

|  | Si | No | Desconocido |
| --- | --- | --- | --- |
| Levantarse de la cama o silla |  |  |  |
| Sentarse |  |  |  |
| Caminar |  |  |  |
| Acostarse |  |  |  |
| Otros |  |  |  |

Pregunta 4. El dolor que ud o su niño perciben, le impide asistir a su trabajo o escuela?

Si____ NO ____

Si la respuesta es afirmativa.

Cuanto Tiempo Falto?

Menos de 1 semana___

1 Semana.______

2 Semanas______

3 Semanas_______

4 Semanas o mas_____

Pregunta 5. El dolor que Ud o su niño percibe, le imposibilita realizar movimientos en una o más articulaciones?

Si_____ No_______

Si la respuesta es Afirmativa.

Cual o cuales de las siguientes no puede realizar movimientos?

|  | Si | No | Desconocido |
| --- | --- | --- | --- |
| Cuello |  |  |  |
| Hombros |  |  |  |
| Codos |  |  |  |
| Muñecas |  |  |  |
| Mano |  |  |  |
| Region Lumbar |  |  |  |
| Cadera |  |  |  |
| Rodillas |  |  |  |
| Tobillos |  |  |  |
| Pies |  |  |  |

Pregunta 6. Ud. o su niño toman algún medicamento para el dolor?

Si_____ NO____

Si la respuesta es afirmativa.

Cual o cuales de los siguientes medicamento está tomando?

| Medicamento | Si | No | Desconocido |
| --- | --- | --- | --- |
| Acetomenofen |  |  |  |
| Ibuprofeno |  |  |  |
| Aspirina |  |  |  |
| Novalgiina |  |  |  |
| Diclofenac |  |  |  |
| Esteroides |  |  |  |
| Naproxeno |  |  |  |
| Otros medicamentos |  |  |  |

Otros medicamento: especificar el nombre del medicamento_______

Referido a Unidad de salud?

Si____ NO________

Si la respuesta es afirmativa.

Razón por la cual se remitió?________________

Llenar esta parte si la encuesta fue llenada en la unidad de salud y/o personal de salud ,

1. El participante presenta edema en una o más articulaciones ?

| Edema | Si | No | Desconocido |
| --- | --- | --- | --- |
| Cuello |  |  |  |
| Hombros |  |  |  |
| Codos |  |  |  |
| Muñecas |  |  |  |
| Mano |  |  |  |
| Region Lumbar |  |  |  |
| Cadera |  |  |  |
| Rodillas |  |  |  |
| Tobillos |  |  |  |
| Pies |  |  |  |
| Otros |  |  |  |

Si la respuesta es afirmativa en otros: Especificar________________

2. El participante presenta deformidad en una o más articulaciones?

| Deformidad en Articulaciones | Si | No | Desconocido |
| --- | --- | --- | --- |
| Cuello |  |  |  |
| Hombros |  |  |  |
| Codos |  |  |  |
| Muñecas |  |  |  |
| Mano |  |  |  |
| Region Lumbar |  |  |  |
| Cadera |  |  |  |
| Rodillas |  |  |  |
| Tobillos |  |  |  |
| Pies |  |  |  |
| Otros |  |  |  |

Si la respuesta es afirmativa: Especificar ________________

3. El particpante presenta tenosinuvitis en una o mas articulaciones?

| Tenosinuvitis | Si | No | Desconocido |
| --- | --- | --- | --- |
| Cuello |  |  |  |
| Hombros |  |  |  |
| Codos |  |  |  |
| Muñecas |  |  |  |
| Mano |  |  |  |
| Region Lumbar |  |  |  |
| Cadera |  |  |  |
| Rodillas |  |  |  |
| Tobillos |  |  |  |
| Pies |  |  |  |
| Otros |  |  |  |

Si la respuesta es afirmativa: Especificar ________________
